# Knowledge and practice of condom use as well as perceived barriers among street adolescents in Cameroon

**DOI:** 10.4102/sajhivmed.v17i1.479

**Published:** 2016-11-03

**Authors:** Samuel Nambile Cumber, Joyce M. Tsoka-Gwegweni

**Affiliations:** 1 Department of Nursing and Public Health, College of Health Sciences, University of KwaZulu-Natal, South Africa

## Abstract

**Introduction:**

Street children in Cameroon are adolescents, vulnerable to sexually transmitted infections (STIs) and HIV and/or AIDS. The level of knowledge and practice of condom use among this population is unknown.

**Objective of the study:**

To assess the knowledge, practice and barriers to condom use in Cameroon.

**Materials and methods:**

The study was an analytical cross-sectional survey conducted in 2015. Questionnaires were administered to street children in a quiet location. Recruitment was made using the snowball technique with the help of peers.

**Results:**

More than 90% of participants knew of condoms, but only about 6% reported to have used a condom during their last sexual encounter. Most of the participants did not know that condoms could prevent HIV; only a few (15.5%) knew about this.

**Conclusion:**

Street adolescents in Cameroon seem to know about condoms, but have insufficient information on the importance of their regular use. The main barriers for the low practice of condom use reported by this population were the following: condoms hinder sexual pleasure; are costly; and it is embarrassing to buy, use or propose to use a condom.

## Introduction

Street children are mostly adolescents in most African countries.^1^ They are often children under the age of 18 years living in difficult circumstances on the streets as has been identified by the United Nations Children’s Fund (UNICEF).^2,3^ Concerns have also been drawn by UNICEF regarding their growing numbers on urban streets in low- and middle-income countries around the world.^2,3^ The existing estimate of street children in urban cities remains that of UNICEEF, which suggested that there are tens of millions of street-based children in urban cities and their number continues to rise more rapidly in low- and middle-income countries around the world.^2,3^ The increase of street children in these countries may be because of rapid urbanisation, migration, population growth, the HIV and/or AIDS epidemic and other health challenges of parents.^3,4^

While on the streets, they go around without any form of proper adult supervision; thus, the framework of a family is absent – a framework which is believed to foster healthy development and growth in children. While on the streets, these children are pushed by circumstances to engage in any risky form of activity in order to survive life on the streets.^3,4^ As most of them lack basic education and skills, they are more likely to beg; tell lies; steal; engage in car washing, shoe shining, plate washing in restaurants, scavenging and other activities for their survival.^3,4,5^

In Cameroon, their number is unknown, but they can be seen wandering the urban streets in dirty torn clothes, with some looking pale and sick.^4,6,7^ Despite their appearance in urban cities in Cameroon, no adequate supervision or support from the community or government has been given to them; thus, they are completely left to survive on their own, despite their vulnerability on the streets.^4,6,7^ The population of young people under the age of 18 years is estimated to be 9 573 563, almost half of the country’s total population.^7^ Total yearly deaths in Cameroon is estimated to be 117 415 and daily deaths to be 564.^7^ The estimated total life expectancy at birth for both sexes in Cameroon is 54.4 years, 53.5 years for males and 55.3 years for females.^7^ However, despite the poorly estimated figures given above, the Cameroon health system has no published data on the health of street children and there are no existing policies which give street children any priorities when they seek healthcare.^6,7^ Furthermore, the public healthcare services in Cameroon are not freely available to all because of its service fee policy without which one cannot receive primary healthcare in both private and state-owned healthcare facilities across the country.^6,7^

The policies towards street children and the high hospital cost for treatment all weigh much more on street children who are faced with the harsh realities on the streets in Cameroon.^6,7^ They are more likely to experience sexual abuses and physical violence on the streets at night, exposing them to the risk of acquiring sexually transmitted infections (STIs), and pregnancy among females.^6,7^ Furthermore, to survive on the streets, many of them engage in risky behaviours such as sex work, substance abuse and multiple sexual partners, among others, which increase their risk of acquiring STIs including HIV and/or AIDS.^5,6^ We reported elsewhere that more than 80% of street children, in the three main cities of Cameroon, reported to have STIs in the past.^8^

Condoms are well-known contraceptives that are used during sexual intercourse to reduce the probability of pregnancy and the spread of STIs including HIV and/or AIDS.^9,10,11^ Condoms have gained more importance in the prevention of the spread of AIDS; yet a large section of the population of street children still have unprotected sexual intercourse and continue to have multiple sexual partners.^9,10,11^ One of the biggest problems with the use of condoms is that they are not used consistently, especially among street children.^9,10,11^

Perceived barriers to condom use by street children may include factors such as high prevalence of rape, the unfavourable economic position of street children and their inability to insist on the use of condoms, which hampers their ability to negotiate during sex as the timing of sexual intercourse and the conditions under which it occurs are all to the disadvantage of street children.^9,10,11^

Personal factors, individual perceptions, lack of efficiency, notions, constraints and condom-related problems are likely to influence condom use among street children; this especially affects girls, who account for 25% of the street children, as many of them take to prostitution as a means of survival.^9,10,11^

Most of their clients do not want to use condoms and offer additional money for unprotected sexual intercourse; thus, they end up with common STIs, HIV or become pregnant for which they lack the means to seek medical aid.^9,11^

Acute alcohol consumption and the use of other drugs by street children significantly predicts their perceived likelihood of having sexual intercourse without condoms.^12,13^ Both a feeling of emotional closeness and an increased sexual arousal are associated with the consumption of psychoactive substances.^12,13^

The aim of this study is to document the knowledge and practice of condom use among street children in three Cameroonian cities. Specific objectives are to assess their knowledge regarding the use of condoms, describe the practice of condom use and identify barriers to condom use.

In Cameroon, little is known about condom use among street children, and the barriers to condom use among them are unknown as no study has published such information.^7^ This information is very important for the government and all stakeholders in order to address the barriers faced by these children regarding condom use.

## Materials and methods

This study was part of a comprehensive cross-sectional survey conducted during a 3-month period from 01 January to 30 March 2015 to investigate the level of knowledge and practice of condom use as well as perceived barriers among 399 street children (320 boys and 79 girls), who were below the age of 18 years during the time of the study and resided in Bamenda, Douala or Yaoundé urban city in Cameroon.

The study used the phrase ‘street children’ in order to refer to children living on the streets in urban cities, and considers a street child to be any child, boy or girl, below the age of 18 years, who has taken to the streets (including wastelands, unoccupied dwellings and unfinished buildings) as their habitual abode and source of livelihood without proper adult supervision. Because the participants had no identity cards to prove their age, the study relied only on self-reported ages, which was reported orally by the participants. The study selected three urban cities which accounted for accommodating large numbers of street children. Bamenda is the most populated English-speaking city in Cameroon, Douala is the economic capital of Cameroon and Yaoundé is the administrative capital of the Republic of Cameroon. Douala and Yaoundé are French-speaking cities and are the most populated among the French-speaking cities in Cameroon.

In the absence of data on street children in Cameroon, a 50% prevalence value was used for calculating the sample size, using the following formula:

$$n=\frac{Z^{2}*P(1-P)DEFF}{d^{2}}$$ [Eqn 1]

where *n* = target sample size; *Z* = statistics for a level of confidence (here *Z* = 1.96, i.e. for a 95% confidence interval); *P* = estimated proportion of street children; *d* = absolute precision, that is, the width of the confidence interval to be 5% (0.05); and *DEFF* (design effect): used a clustered-randomised study where the different cities are the primary sampling units. The same number of participants was used in the different cities of the study, giving a design effect to be 1.

Therefore:

$$n=\frac{1.96^{2}(0.5)(1-0.5)(1)}{0.05^{2}}=385$$ [Eqn 2]

Also, based on assumption, about 5% of the questionnaires would be wrongly filled in or incomplete. For this reason, 20 more questionnaires (i.e. 0.05*385) were added, giving a total of 405 targeted participants (street children) for this study.

Participation in the study was voluntary. All participants were 12–17 years of age at the time of the study and must have been living on the streets for not less than 1 month prior to the onset of this study. The pilot study showed more family contact with children less than a month on the street. This does not mean that those who are less than 1 month on the street are not street children, but the authors believed those who are more stable and have accepted the street to be their new home will have more to contribute towards the study objectives.

Recruitment and enrolment took place at the local Catholic Church facilities, which provided a quiet and natural location for data collection. Data collection was done by administering questionnaires to the participants because it is believed that most of the street children cannot read or write. The questionnaires were administered using Pidgin English and French (Pidgin English is a language that is widely spoken in West Africa). The children were first contacted at identified gathering points for street children (e.g. bus stations, railway stations, market and city centres, and in front of movie houses). The questionnaires were administered by six research assistants who were given a 2-day training course to ensure that they all understood the questionnaire on the same level. Questions asked included demographic details, knowledge regarding condom use, perceived barriers to condom use, history of condom use and the source of condoms.

A non-probability technique (snowball sampling) was used to get participants with the help of peers on the street. This is a process whereby each recruited participant led the researcher to other participants. Snowball was used because of the highly mobile lifestyle of street children. In total, 405 street children were recruited; however, six participants were excluded before analysis because of incomplete information, resulting in 399 participants.

To protect the identity of the participants as all were minors, no names were written on the questionnaires; however, unique numbers were assigned to each participant for the purpose of the research only. Only the primary researcher had access to the collected data.

Obtaining permission from parents and/or guardians in the case of street children was not possible. Thus, only written informed consent form was obtained. The children were first approached by the research team at their meeting point and the entire study was explained to them including their rights and the freedom to withdraw from the study at any time they feel uncomfortable.

After administering of the questionnaires, food was given to the participating street children but no monetary incentive was provided.

The study received ethical approval from the Biomedical Research Ethics Committee, University of KwaZulu-Natal, Durban, South Africa and the Cameroon Bioethics Initiative.

After data collection and cleaning, data were captured in a Microsoft Excel (2010) spreadsheet and imported to SPSS statistical package version 19 for Windows (IBM Corp., Armonk, NY, USA) for analysis. Descriptive statistics such as frequency distributions and cross-tabulations were used to summarise the data. A normal distribution test was performed to determine whether the data were normally distributed or not. Chi-squared test of association was used to assess whether there was any association between region and other categorical variables related to condom use. All the chi-squared tests were conducted at 5% significance level; thus, a *p*-value less than 0.05 meant that there was some significant variation in the variable of interest.

A pilot study was conducted using a preliminary version of the authors’ self-designed questionnaire. The questionnaire was tested on a small sample of street children in a different city, after which the authors improved on the training given to research assistants and amended some questions to be more simple.

The study has many strengths including the fact that up to 79 girls were recruited, making it the only study to have recruited this number of street girls in Cameroon. Also because of the large sample size (399 street children), six research assistants were employed for this study.

The main weakness of the study was that it relied mainly on self-reported data and it was not possible to verify the responses provided by participants about their present living conditions and family background. Also, there was no identification papers presented to determine their actual age. The lack of sufficient finance made it difficult to collect data in all 10 regions of Cameroon.

## Results

### Demographic information

In total, 399 adolescents were interviewed. Of the sample, 320 (80.2%) were boys and 79 (19.8%) were girls. The age group 15–17 years made up 77.7% of participants, and was evenly distributed in all three cities. As presented elsewhere by Cumber and Tsoka-Gwegweni^8^, the age range 12–17 had a mean age of 15.4±1.27 years. Over 80% of participants reported they were Christians, and less than 10% said they were either Muslims or traditional and/or non-religious, with Yaoundé having the highest proportion of Muslims. Regarding the educational status of the participants, 21.3% had never been to school, 77.4% had primary education and 1.3%, from Douala, studied up to secondary school. The reason for school dropout was reported as family poverty, hatred for school, bullying and maltreatment by teachers. All the demographic variables analysed revealed significant differences between the cities, except for the age groups of participants. More than 80% of participants reported having suffered from a STI, with the highest percentage coming from Douala and Yaoundé. All participants reported being sexually active (Table 1).

**TABLE 1:** The demographic characteristics of participants by city.

Variable	Characteristics	Bamenda†		Douala‡		Yaoundé§		Total¶		*p*
		*N*	%	*N*	%	*N*	%	*N*	%	
Sex	Male	108	86.4	101	73.7	111	81	320	80.2	0.035
	Female	17	13.60	36	26.3	26	19	79	19.80	-
Age	12–14 years	29	23.2	31	22.6	29	21.2	89	22.3	0.919
	15–17 years	96	76.8	106	77.4	108	78.8	310	77.7	-
Religion	Christian	99	79.2	121	88.3	111	81	331	83	0.016
	Islam	9	7.2	13	9.5	14	10.2	36	9	-
	Tradition and/or none	17	13.6	3	2.2	12	8.8	32	8	-
Educational level	No formal education	40	32	18	13.1	27	19.7	85	21.3	0.001
	Primary	84	67.2	115	83.9	110	80.3	309	77.4	-
	Secondary	1	0.8	4	2.9	0	0	5	1.3	-
Reason for school dropout	No money	40	32	113	82.5	59	43.1	212	53.1	0.000
	Did not like school	56	44.8	11	8	61	44.5	128	32.1	-
	Bullied in school and/or teachers not nice	29	23.2	13	9.5	17	12.4	59	14.8	-
	Sexually active	125	100	137	100	137	100	399	100	-

† *n* = 125;

‡ *n* = 137;

§ *n* = 137;

¶ *n* = 399.

### Knowledge of condom use by city

Table 2 presents results of knowledge regarding condom use in the three cities of Cameroon. It should be noted that more than 90% of participants said they had knowledge of condoms even before ever having sex, but only about 10% reported being aware of the importance of regular use of condoms. Also, only 15.5% of participants knew that condoms could prevent HIV and/or AIDS.

**TABLE 2:** Knowledge regarding condom use by city.

Statement	Category	Bamenda†		Douala‡		Yaoundé§		Total		*p*
		*N*	%	*N*	%	*N*	%	*N*	%
Aware of the importance of condoms	Correct	13	10.4	16	11.7	13	9.5	42	10.5	0.6362
Know of condoms before sex	Question	125	100	127	92.7	129	94.2	381	95.5	0.0115
Condoms protect against HIV	Correct	27	21.6	20	14.6	15	11	62	15.5	0.0553
Condoms protect against STDs	Correct	99	79.2	109	79.6	113	82.5	321	80.5	0.7586
Condoms can be reused	Incorrect	98	78.4	79	57.7	75	54.8	252	63.2	< 0.0001
Condoms must be checked for leaks	Correct	83	66.4	64	46.7	57	41.6	204	51.1	0.0001
Putting on a condom before penetration is a must	Correct	84	67.2	35	25.6	51	37.2	170	42.6	< 0.0001
Unroll condom before putting on the penis	Incorrect	95	96	91	66.4	87	63.5	273	68.4	0.0777
Condoms can be used with lubricants	Correct	113	90.4	115	83.9	111	81	339	85	0.0968
Condoms should be put on only before ejaculation	Incorrect	102	81.6	84	61.3	90	65.7	276	69.2	0.0010
Withdrawal of penis immediately after ejaculation	Correct	103	82.4	45	30.7	41	29.9	189	47.4	< 0.0001

Note: The category section gives results for street children who responded either correctly or incorrectly for that particular question (the percentage).

† *n* = 125;

‡ *n* = 137;

§ *n* = 137.

More than 60% of participants thought that condoms could be reused, with a significantly higher frequency in the city of Bamenda. About 69.2% knew that condoms should be put on before ejaculation, with the highest frequency in Bamenda (81.6%). Also 47.4% of participants reported that the penis should be withdrawn immediately after ejaculation when using a condom, with still a significantly higher frequency in the city of Bamenda (82.4%).

### Practice of condom use among street children stratified by city

**FIGURE 1:**
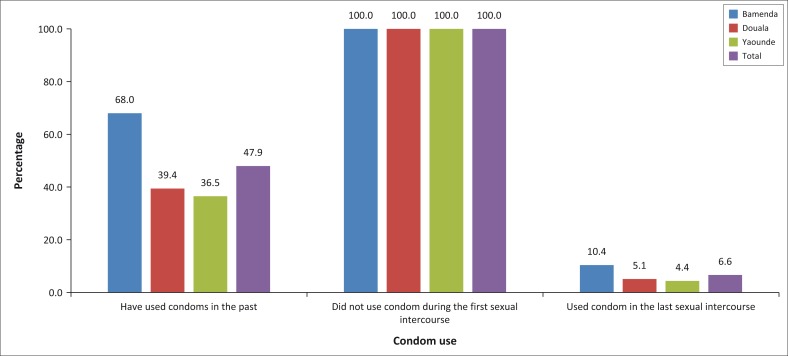
History of condom use among street children stratified by region. The figure shows percentages based on each city, for example, 68% of participants only in Bamenda have used condoms in the past.

All the participants reported not having used a condom during their very first sexual intercourse (Figure 1). A similar response was received when participants were asked about condom use during their last sexual activity, as only 6% of participants reported to having used a condom during their last sexual intercourse. The responses were much lower in Douala and Yaoundé compared to that in Bamenda. Less than half of the participants reported to ever having used a condom in the past as shown in Figure 1.

**FIGURE 2:**
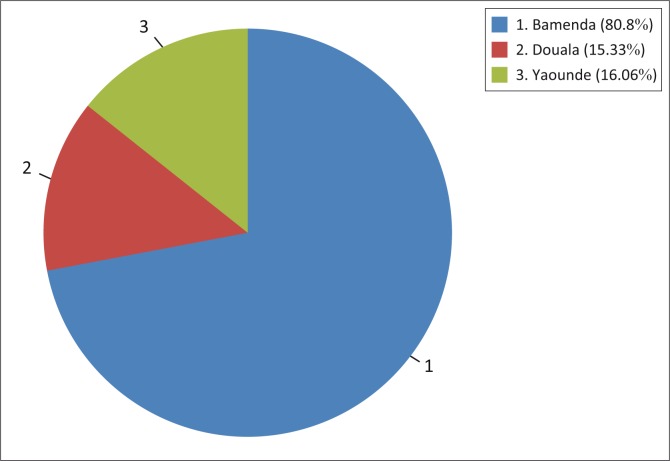
Frequencies of participants who never used condoms.

More than half of the participants reported using condoms, although it was not regular. Higher frequencies were reported in Yaoundé and Douala. It should also be reminded that a significant percentage of participants reported to have never using a condom before (Figure 2).

### Perceived barriers to condom use

Different reasons were given by participants for not using condoms. A considerable proportion of the participants mentioned the discomfort and painful nature of the condom as a reason of not using it.

Some participants reported that they were shy to ask partners to use a condom, while the very few who reported not being shy to buy or use condoms were more concerned or were afraid of the aggressive reaction of their partners if they proposed the use of condoms.

Participants also reported some common reasons identified as barriers which contributed to not using condoms, such as: ‘I did not think about condoms’, ‘my partner did not care’, ‘I do not have HIV and/or AIDS’, ‘condoms hinder sexual satisfaction’, ‘I had no condom’ and ‘I was drunk and forgot’, among others (Table 3).

**TABLE 3:** Perceived barriers to condom use.

Barrier	Bamenda†		Douala‡		Yaoundé§		Total		*p*
	*N*	%	*N*	%	*N*	%	*N*	%	
**Reasons**									
Condoms hinder sexual satisfaction	115	92	133	97.1	130	94.9	378	94.7	0.1833
Was with a steady partner	113	90.4	133	97.1	132	96.4	378	94.7	0.0311
Do not have HIV and/or AIDS	115	92	137	100	137	100	389	97.5	< 0.0001
Sex always happens too fast	112	89.6	133	97.1	133	97.1	378	94.7	0.0081
My partner did not care	112	89.6	134	97.81	134	97.81	380	95.2	0.0017
Did not think about using condoms	112	89.6	130	94.9	129	94.2	371	93	0.1972
Had no condom with me at the time	108	86.4	135	98.5	134	97.8	377	94.5	< 0.0001
Feel embarrassed to buy condoms	111	88.8	107	78.1	110	80.3	328	82.2	0.0597
Feel embarrassed to use condoms	116	92.8	117	85.4	121	88.3	354	88.7	0.1645
My partner got angry for suggesting we use condoms	24	19.2	65	47.5	63	46	152	38.1	< 0.0001
Condoms are painful and discomforting	83	66.4	101	73.7	82	59.9	266	66.7	0.0514
Want to be pregnant	14	11.2	6	4.4	9	6.6	29	7.3	0.0972
Contrary to religious beliefs	50	40	8	5.8	7	5.1	65	16.3	< 0.0001
Was drunk and forgot	94	75.2	131	95.6	132	96.4	357	89.5	< 0.0001
**Frequency of condom use in the past 3 months**									
Regular(every time you had sex)	6	4.8	7	5.1	5	3.7	18	4.5	< 0.0001
Irregular(sometimes you have sex)	18	14.4	109	79.6	110	80.3	237	59.4	-
Never used	101	80.8	21	15.3	22	16.1	144	36.1	-

† *n* = 125;

‡ *n* = 137;

§ *n* = 137.

**FIGURE 3:**
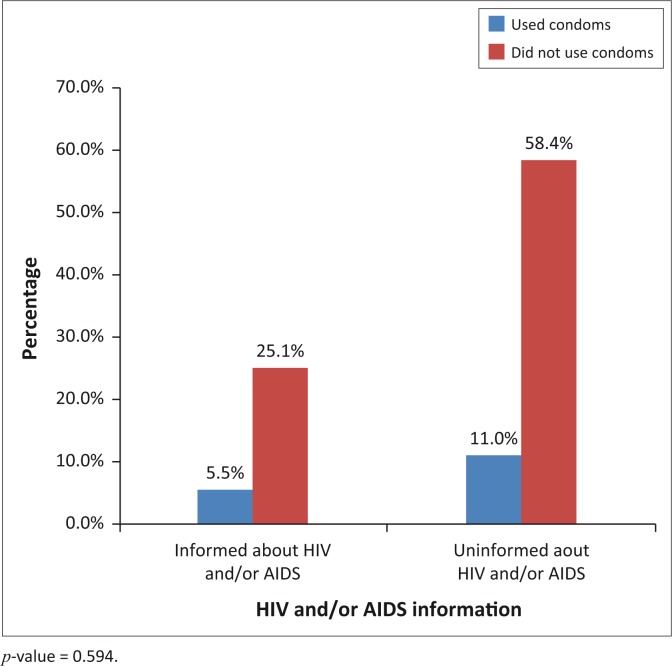
Relationship between information and condom use for prevention of HIV and/or AIDS.

In general, 30% of participants have heard information about HIV and/or AIDS, but only a few of them actually implemented prevention. Furthermore, even the few who had knowledge of HIV and/or AIDS never used the information (like to insist on the use of condoms) to protect themselves (Figure 3).

## Discussion

This study focused primarily on three aspects: the knowledge, practice and barriers, of condom use among street children in three urban cities (Bamenda, Douala and Yaoundé) in Cameroon. Their demographic details, living conditions and risky sexual behaviours were gathered through self-reports rather than document review and/or medical examinations. Self-reports may be regarded as less reliable sources; however, with the challenges surrounding the mobile lifestyle of street children, we believe that our results are a true reflection of their knowledge, practice and perceived barriers in using condoms. This belief is based on the fact that the study results concur with other similar studies in Africa.^3,4,5^ This study also confirmed directly or indirectly that the street presents opportunities for work for these children, despite the adverse circumstances they have to endure on the streets. Thus, the results are similar to those from other studies conducted in Kinshasa, Egypt, Addis Ababa, Ghana and Rwanda.^9,10,11,12,14^

This study further confirmed that street children belong to a group with multiple vulnerabilities and at high risk for HIV infection because of their lifestyle and other risky behaviours such as unprotected sex, sex work, multiple sexual partners, among others, as a means to survive the harsh realities on the streets. Unlike studies from Rwanda,^14^ further data to support public health interventions were beneficial. However, public health interventions have not included street children in Cameroon; thus, there are no existing data on previous public health interventions on this population.^7^

No study conducted in Cameroon had addressed specifically the knowledge, practice and barriers to condom use among street children in Cameroon. For this reason, the Ministry of Public Health in Cameroon has called for papers which can describe the current knowledge, practice and common barriers to the use of condoms among this population.^7^ Because of no current data, this study aims at presenting the situation using a descriptive approach so as to provide data on the present situation, which will enable decision-makers and other stakeholders working with street children to be able to design sustainable interventions to help the growing number of vulnerable street children.

The results reveal that all participants, irrespective of the city setting, had limited knowledge of the importance of condoms. Also a high proportion of respondents (street children) did not know about the correct routes of HIV transmission and this could be one reason for the irregular use of condoms alongside other barriers (such as high cost, shyness and fear, among others) as outlined in the results. Poor knowledge may also be a result of their source of information which includes mainly their peers and other street friends. The results also revealed that all 399 participants reported to be sexually active and some, though mostly girls, engaged in commercial sex with adults in the community without the use of condoms. This suggests that the efforts made by the government and other community-based organisations to fight HIV and/or AIDS in Cameroon are not reaching the population of street children, thereby contributing to the high AIDS mortality among street children in Cameroon hospitals.^7^

The findings of this study showed very low levels of condom use among street children, as well as low levels of knowledge among the adolescents included in the study. Multiple barriers have been highlighted above in the results section.

Additional analyses also revealed that street adolescents with more knowledge on condoms were more likely to have history of condom usage although regular usage scored zero percentage. The percentage of participants who have never used a condom was higher in the city of Bamenda, as compared to the other two cities. This could be because of the strong stereotype behaviour towards persons who freely buy condoms in public places. This is also backed by a considerably higher percentage of participants who reported to be embarrassed buying or using condoms. The percentage could be higher in Bamenda because it is a cultural traditional city with cultural norms very much in place as compared to Douala and Yaoundé which are, respectively, the economic capital and the administrative capital of Cameroon, the two most populated cities in Cameroon and where culture and tradition does not easily come to play a role.^4,6,7^

The results further showed that hindrance of sexual pleasure, feeling embarrassed to buy or use condoms and other reasons were identified by participants as barriers to condom use. These results are similar to other studies conducted on street adolescents in some African countries such as Kinshasa, Egypt, Addis Ababa, Ghana and Rwanda.^3,9,10,11,12,14^ The circumstances that make street children more vulnerable than children who live in homes are similar to those reported in studies from other African countries such as Egypt, Addis Ababa, Ghana and Rwanda, which highlighted scanty knowledge and misconceptions about condoms.^3,9,10,11,12,14^

A study from Egypt and Ghana reported that about half of street children knew that unprotected sex was the primary mode of HIV transmission, but that did not affect their level of engaging in risky sexual behaviours (unprotected sex).^10,12^ Sexual debut was reported to be from 12 years as all participants reported being sexually active, but similar results were reported among street children in other countries, some having had sex even from the age of 8 years.^3,9,10,11,12,14^ Furthermore, cultures in West Africa overlook early sexual intercourse for males, and because of their young ages their initiation in most cases is with prostitutes, which puts them at risk of contracting STIs and HIV because condoms are not being used.^3,9,10,11,12,14^

Future interventions should primarily strengthen the knowledge of street adolescents, target the various age groups as well as gender to obtain a better understanding of their knowledge, practice and perceived barriers to condom use.

## Conclusion and recommendation

The survey showed a clear insight into street children in Cameroon regarding their condom use and barriers to the use of male condoms. The results showed that the street children know about condoms but have insufficient information regarding the importance of regular use of condoms. Although not being able to afford condoms can be seen as a strong barrier, the push and determination to use condoms was not seen as a priority and the government and non-governmental organisations have completely neglected street children during their intervention campaigns. More programmes should target street children specifically in Cameroon.

The authors of this study suggested that condom vending machines and increasing the number of distributing points of free condoms in the community will help ease the stress of buying condoms.

Interventions from all stakeholders should tailor their messages to eliminate barriers or reduce them to a minimum, and this can be achieved through creating stronger and trustful relationships with street children, providing free condoms to them and improving their knowledge regarding what condoms can prevent. Thus, intervention programmes should intensify condom education with street children. The government and non-government agencies should also intensify public campaigns targeting the entire community, promoting condoms as sexual diseases preventive measures because street children do not only have sex with street children but also with people in the community.
